# Medical Students’ Socioeconomic Status and Academic Performance in Medical School

**DOI:** 10.7759/cureus.39875

**Published:** 2023-06-02

**Authors:** Kencie Ely, Gemma Lagasca, Shaun Andersen, Deepal Patel, Edward Simanton

**Affiliations:** 1 Medical Education, University of Nevada Las Vegas School of Medicine, Las Vegas, USA; 2 Office of Medical Education, University of Nevada Las Vegas, Las Vegas, USA

**Keywords:** gpa, mcat, usmle, medical school admissions, financial burden, medical student, academic performance, socioeconomic status, medical school

## Abstract

Background

Students from lower socioeconomic groups tend to underestimate their chances of acceptance to medical school and their likelihood of success once admitted.

Objective

The objective of this study is to determine if socioeconomic disadvantage status is linked to lower medical college admission test (MCAT) scores and academic performance in medical school.

Methods

Using the Association of American Medical Colleges (AAMC) education/occupation (EO) indicator, we compared economically disadvantaged students to students with no financial disadvantage on the MCAT, Phase 1 National Board of Medical Examiners (NBME), United States Medical Licensing Examination (USMLE) Step 1, Phase 2 NBME, and USMLE Step 2 test scores.

Results

Medical students in the disadvantaged group scored significantly lower on the MCAT than students with no financial disadvantage. The disadvantaged group showed a non-significant lower trend in performance until USMLE Step 2.

Conclusions

Applicants from lower socioeconomic backgrounds may perform lower on their MCAT and early medical school benchmark exams, but they appear to catch up with and even surpass their peers on their USMLE Step 2 examination.

## Introduction

Socioeconomic status (SES) is a measure of one’s standing as it relates to their income, education, and occupation [1]. The effect of socioeconomic status (SES) on educational attainment is evident as early as kindergarten and persists through grade 12. Multiple factors contribute to the limitations of low-SES students. Students from low-SES households are less likely to have parental involvement in their education, which has significant effects on self-efficacy and overall performance [2]. Additionally, lower-SES students are often expected to contribute to the household in more ways than their higher-SES counterparts, for example, by holding a part-time job after school or babysitting younger siblings. These factors, compounded with parental expectations that a college degree is not required, can discourage students with low SES from pursuing a bachelor’s degree [3]. For those students who do move forward into higher education, the burdens in place during their high school career continue to loom over them. These barriers influence students of low SES, so they are less likely to finish their degree, let alone pursue a graduate degree [3]. 

Students who overcome these barriers and decide to pursue a medical degree are once again met with barriers that their high-SES counterparts do not have to face. Juggling familial obligations and their undergraduate studies has the potential to hurt their grade point average (GPA) and take time away from studying for the Medical School Admissions Test (MCAT), which are both important metrics during the medical school admissions evaluation process [4-5]. Having a low GPA and MCAT score can limit a student's ability to stand out from their peers and, in some cases, automatically disqualify an applicant if they do not meet the school’s minimum threshold. Other differences, such as less social support from peers and mentors, have led many low-SES students to believe that they do not belong in the medical field and are unable to succeed [6].

Despite improvements in inclusivity measures, students from low-SES backgrounds continue to be underrepresented in medicine [7,8]. This is concerning given the data that shows physicians who come from a low-SES background are more likely to have attitudes that focus on patient-centered care and positive outcomes [9]. Between 1987 and 2005, the majority of medical students came from high-SES families [1,9]. To help medical schools diversify their socioeconomic representation, the Association of American Medical Colleges (AAMC) created an SES indicator starting with the 2014 application cycle. According to the AAMC, 23%-24% of all applicants during the 2018-2021 application cycle were classified as SES-disadvantaged, and of those applicants, 20%-21% matriculated into medical school [10]. Studies have been done to understand why students from low-SES backgrounds are less represented in medicine than their higher-SES counterparts [6]. Several of these factors are related to the aforementioned emotional and financial barriers they face. 

Once in medical school, students from low-SES backgrounds have to overcome the belief that they cannot succeed while also dealing with the typical challenges most medical students face. Some of the most significant issues include financial obligations, the overwhelming workload, pressure to succeed, relationships, and work-life balance [11]. These mental, emotional, and financial hurdles could further impair a student from low SES in succeeding academically and contribute to their belief they will not succeed [12]. As previously stated, students from low-SES backgrounds perform lower on exams before medical school. However, data is limited about their performance while they are in medical school [5]. Throughout medical school, students complete preclinical (Phase 1), clinical (Phase 2), and United States Medical Licensing Examination (USMLE) Step 1 and USMLE Step 2 exams. Phase 1 and Phase 2 examinations consist of questions from the National Board of Medical Examiners (NBME) and are required for each medical student to demonstrate mastery over the content they are learning and to prepare them for USMLE exams. Allopathic students are required to take the USMLEs to obtain medical licensure in the United States. One study done at the University of California Davis School of Medicine showed that students who identified as socio-economically disadvantaged performed worse on the USMLE Step 1 and Step 2 exams compared to their non-economically disadvantaged counterparts [13,14]. Although this study assesses performance, it was only conducted at a single institution and did not use the AAMC Education/Occupation (EO) indicator to assess economic disadvantage. We sought to determine if this trend was present among a different student population when implementing the EO indicator used by the AAMC. Our study aims to determine what differences, if any, are present between the academic performance of low-SES students and high-SES students at the Kirk Kerkorian School of Medicine (KSOM) at the University of Nevada, Las Vegas.

## Materials and methods

Subjects and setting

KSOM is a public medical school located in Nevada’s largest city, Las Vegas. It is the first allopathic medical school in Southern Nevada and was founded to address the long-standing physician shortage in the area. In 2010, Nevada ranked 45th in the country in the number of physicians per 100,000 people [15]. This shortage impacts community members at large, who are forced to wait weeks to get appointments to see primary care physicians, with the impact affecting those coming from low SES the most [16]. In turn, since the inception of the school in 2017, an emphasis has been placed on admitting students who will remain in the Las Vegas Valley after graduation. The admissions team has attempted to accomplish this goal by selecting students who are from the community, as it has been shown that students who have personal ties to the patient population they are serving are more likely to practice in those communities [14]. In addition, the KSOM mission emphasizes values such as inclusion and community health. To address these values, our student body has been curated to reflect our community’s diversity, including students from various SES backgrounds. In a statement provided by the 2022 Admission Committee Director, Dr. Gasper de Alba states: “The Kirk Kerkorian School of Medicine Admissions Committee Handbook is provided to each member of the committee and is reviewed at the beginning of each admissions cycle. The Policy on Student Selection states the following among desirable characteristics for student selection: first-generation college attendees, economically disadvantaged, and educationally disadvantaged (Title I high school).” With these criteria in mind, our institution’s large number of students from low SES makes it an ideal location to measure the effects of socioeconomic disadvantage (SED) in medical school. 

KSOM students take a series of system-based block exams comprised of retired NBME questions during their Phase 1 curriculum. Students who fail only one or two NBME block exams are given the opportunity to remediate at the end of each semester using a cumulative final exam. Students are allowed to fail a maximum of three NBME block exams before being put on academic probation, where they are required to complete an extensive remediation process before being allowed to proceed into the next phase. To proceed to clinical rotations, all KSOM students must pass their USMLE Step 1 examination. The Phase 2 curriculum is measured through NBME shelf exams, which are taken twice to give students two chances to achieve their desired scores. In contrast to many other medical school shelf exam schedules, KSOM requires that all shelf exams be taken within one week. The first set of shelf exams is taken at the midpoint of their Phase 2 curriculum, and the second set is taken again at the end of their Phase 2 curriculum. After this phase, their highest scores are retained. The Phase 1 and Phase 2 NBME exam scores recorded are the cumulative average of all exams taken during their respective phases. The current KSOM attrition rate is less than 0.5% out of the five classes that have matriculated since 2017, as compared to the average attrition rate of all allopathic schools since 1997, which is 3.2% [16]. 

Data sources

Data for this study were drawn from KSOM databases in accordance with the approved International Review Board (IRB) protocol number 1030906-1, entitled “School of Medicine use of program evaluation data for research,” dated April 3rd, 2017. The AAMC education/occupation (EO) indicator classifies applicants into five ordered groups (EO1 through EO5, see Appendix A). The groups are categorical aggregates of parental education and occupation for which AAMC identifies EO1 and EO2 as economically disadvantaged. To increase the power of our study, we combined first-, second-, third-, and fourth-year KSOM students who fell into the EO1 or EO2 groups. The EO1 and EO2 groups were then compared to students with no financial disadvantage on the following academic measurements: MCAT (most recent attempt), Phase 1 NBME (average of all first attempts), USMLE Step 1 (first attempt), Phase 2 NBME (average of all highest attempts), and USMLE Step 2 (first attempt). The mean and standard deviation were calculated for each to determine differences between groups. For each measurement, we analyzed and recorded each student’s performance per exam and ranked students into percentiles within their respective cohorts. The group percentile was determined by averaging exam performance. An unpaired t-test was then used to determine statistical significance. An alpha of 0.05 was used for significance in this study. All tests for significance are two-tailed.

## Results

Table 1 shows the descriptive statistics (mean, SD, P-value) of the Economic Disadvantage and No Economic Disadvantage groups for the MCAT, Phase 1 and Phase 2 NBME, and USMLE Step 1 and Step 2 performance to determine if either group had a significant variance in the test scores. Different exam companies distribute each examination and therefore vary their score ranges and average exam performance. The MCAT exam scores range from the lowest possible score of 472 to the highest possible score of 528. Phase 1 and Phase 2 NBME exams range from a possible score of 0 to 100. USMLE Step 1 and Step 2 exams range from 1 to 300. 

**Table 1:** Descriptive statistics of population MCAT: Medical college admission test, USMLE: United States medical licensing examination, NBME: National board of medical examiners

Table 1. Descriptive Statistics of Population					
Examination		N	Mean	Std. Deviation	P-value
MCAT	Economic Disadvantage	83	507.55	4.674	< .001>
	No Economic Disadvantage	157	510.27	5.116	
Phase 1 NBME	Economic Disadvantage	69	82.00	0.052	0.4546
	No Economic Disadvantage	110	82.58	0.045	
USMLE Step 1	Economic Disadvantage	67	228.81	15.595	0.4645
	No Economic Disadvantage	105	230.60	15.685	
Phase 2 NBME	Economic Disadvantage	45	77.37	6.478	0.8110
	No Economic Disadvantage	65	78.11	5.963	
USMLE Step 2	Economic Disadvantage	41	247.63	13.306	0.5408
	No Economic Disadvantage	62	245.87	14.875	

Over one-third of the students whose test scores were collected fell within the disadvantaged SES group. Medical students in the socio-economically disadvantaged group had statistically significantly lower MCAT scores than students with no financial disadvantage (mean = 507.55 vs. 510.27, p < .001). 

**Figure 1:**
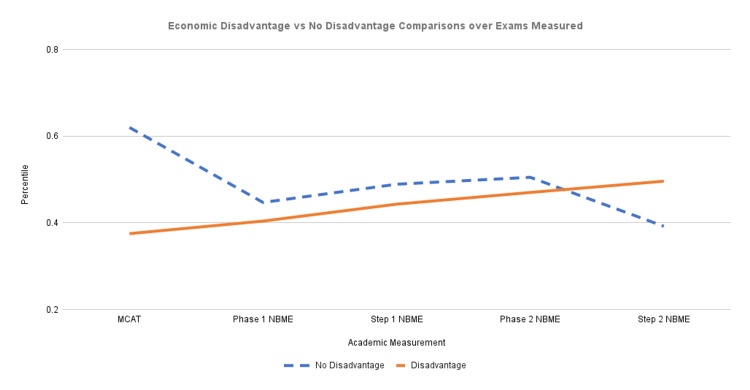
Economic disadvantage vs. No disadvantage comparisons over exams measured

To identify trends in the data for the economic disadvantage and no economic disadvantage groups, students were assigned a percentile amongst their entire group for their particular performance per exam. Then each student’s respective percentiles were distributed into their appropriate economic groups. The average percentiles of the economic disadvantage and no economic disadvantage groups were calculated for every exam. Differences in these averages were assessed for statistical significance using an unpaired t-test with an alpha set to 0.05. Average group percentiles are depicted in Figure 1, with numerical values and levels of significance indicated in Table 2.

**Table 2:** Average percentiles corresponding to Figure 1 MCAT: Medical college admission test, NBME: National board of medical examiners

	No Disadvantage	Disadvantage	p-Value
MCAT Percentile	0.62	0.375	< 0.0001
Phase 1 NBME Percentile	0.447	0.404	0.403
Step 1 NBME Percentile	0.489	0.443	0.462
Phase 2 NBME Percentile	0.505	0.47	0.852
Step 2 NBME Percentile	0.392	0.496	0.647

Socioeconomic disadvantage was negatively associated with MCAT performance. This value was the only measurement that showed statistical significance. Of the remaining benchmarks collected, students from the disadvantaged SES group trended lower than their counterparts until USMLE Step 2; at that metric, their performance increased (though these results were found to have no statistical significance). 

Average group percentiles for the No Economic Disadvantage group and the Economic Disadvantage group for each test were calculated and used to create Figure 1.

Table 2 shows that the biggest difference in percentiles between no economic disadvantage and economic disadvantage was in the MCAT examination scores, with the no economic disadvantage group at a higher average percentile. The no economic disadvantage group continued to have a higher average percentile in Phase 1, USMLE Step 1, and Phase 2 examinations. In the USMLE Step 2 examination, the economic disadvantage group had a higher percentile than the no economic disadvantage group. 

## Discussion

The findings of this study show significant differences in MCAT performance in the economically disadvantaged group compared to their counterparts. Furthermore, although not statistically significant, students from lower SES tend to perform worse on other benchmark exams leading up to the USMLE Step 2 exam than their counterparts. However, some students scored even higher than their peers on this final medical school examination. If economic disadvantage plays a role in student academic performance, then this finding indicates that during medical school, lower SES students were able to overcome some of the academic disadvantages they faced in the early phases of their medical education. Similar results were seen in a study conducted by Jerant et al., who studied medical universities in California [13]. One hypothesis to explain the events that lead low-SES students to catch up to their peers’ centers around the standardization of the medical curriculum within KSOM. For example, each student has equal access to the same material, faculty, and resources. At KSOM, Phase 1 students are given a two-year subscription to a standardized question bank and board exam lecture series. Additionally, academic support services and upperclassman tutors are available to all students. These standardized resources may address one of the main barriers keeping low-SES students from higher performance as undergraduates. If they had consistent and equal access to learning resources, then they may not show deficits in their GPA and MCAT scores. However, as previously stated, the inequity in resources still exists when applying to medical school and can be detrimental to a student’s GPA and MCAT score when applying [4,5]. This topic calls into question the criteria that are used for medical school admissions. It is unrealistic to completely forgo the use of exam performance to help schools evaluate applicants. However, if a low-SES student meets the minimum thresholds, should they be punished for not having access to the same resources and therefore having lower scores on entrance exams compared to their peers? Do alternative measures exist that can be evaluated to determine a person’s potential success in a rigorous environment like medical school? Although a holistic admissions process has taken steps to evaluate a student’s success, there are additional measures that could be used to assess what resources a student had access to while preparing for these exams as well as their aptitude for perseverance in challenging situations.

Additional measures for admission

To determine if a low-SES student will be able to catch up to their peers in medical school, an additional metric should be evaluated. Literature reveals that a person’s mindset is linked to higher academic achievement [17]. One tool that can be used to evaluate a person’s mindset is the Grit scale created by Angela Duckworth [18]. The Grit scale is an 8- and 12-item scale that asks the reader to rank their agreement with several statements concerning the consistency of interests and perseverance of effort on a Likert scale. It has been found that a person’s “grittiness,” in combination with other multifactorial metrics, has the potential to shed light on a person’s ability to succeed in high-stakes situations despite setbacks. Individuals who possess grit tend to be resilient, self-controlled, and ambitious. Studies conducted at chiropractic, physical therapy, and dental schools found that grittier students performed better on their standardized exams and concluded that the grit scale could be used as an additional non-academic measure for admissions [19-21]. We believe that low-SES students who may not have performed as well on their entrance exam as their high-SES counterparts would benefit from an additional evaluation, such as the Grit scale, to assess their potential ability to perform on exams as they traverse through their medical education. 

Interventions

If the reason medical students are able to catch up to their peers by USMLE Step 2 is due to the standardization of medical education, it is possible that earlier identification and intervention would help to alleviate the discrepancies in a medical student’s academic career sooner. One such intervention is the increased utilization of mentorship programs that can help guide premedical students as they attempt to explore their interest in the medical field and eventually navigate the application process. The positive effects of mentorship are well documented across fields and specialties and are well utilized once students are in medical school [22-23]. Undergraduate institutions should prioritize reaching out to pre-medical students from low-SES backgrounds to encourage the utilization of such programs. 

Formal support organizations, such as a post-baccalaureate premedical pipeline program aimed specifically at students from underrepresented and economically disadvantaged backgrounds, could help alleviate some of the incongruities between higher and lower SES students. One such program, MEDPREP, exists and has great success at helping disadvantaged students gain the tools necessary to matriculate into medical school and subsequently become practicing physicians [24]. During this program, students were asked to complete additional science courses to improve their GPA, as well as professionalism and study skills workshops to prepare them for the rigors of professional school. It is unknown how the students from the MEDPREP program performed during their time in medical school; however, of the 525 students who have matriculated through the program thus far, 79% became practicing clinicians. Additional information about these students’ performance during medical school is needed to substantiate the claim that a program like this helps to level the playing field between economically advantaged and disadvantaged students while in medical school. However, MEDPREP and other post-baccalaureate programs incur a significant cost, upwards of $30,000, at some institutions [25-27]. 

There are many third-party resources that offer students application review, personal statement editing, and MCAT preparation courses with promises to “increase your score, guaranteed.” However, to our knowledge, no studies have been conducted to determine if students who utilize these services are accepted into a medical program at a higher rate than students who do not utilize these resources. Furthermore, these services tend to cost as much as $3,000, limiting their benefits to students of low SES. 

Financial burden

Despite the existence of these programs, they come with a financial burden that perpetuates the aforementioned barriers to equal opportunity for disadvantaged students. These programs can further widen the gap between low- and high-SES students since many students who require the use of these programs to make them competitive applicants already have financial obligations and burdens that their high-SES counterparts do not have. In evaluating the efficacy of potential interventions, it is imperative that the downstream and unattended effects are carefully analyzed. Several of the post-baccalaureate programs addressed the cost of attendance concern by granting scholarships to some of the attendees [28]. However, these scholarships were not all-encompassing, and they still omitted many students in need of financial reprieve. 

Assistance with identifying and securing scholarship donations would help to decrease the financial burden that low-SES students face. Despite the abundance of scholarships available, accessing them can be cumbersome. Having to complete another extraneous step that high-SES students do not is a barrier in and of itself, but it can also be difficult to wade through the scholarship opportunities without guidance. More student-specific assistance from financial aid administrators would take pressure off of students in need of these scholarships. By helping to comb through the scholarships available, low-SES students can spend more time crafting their scholarship essays and less time determining whether they are eligible for the scholarships. Indeed, programs exist that filter through scholarships using an algorithm; however, these programs also come at a cost that can deter students from utilizing them. Scholarships based on merit have also been found to be underutilized by students of low-economic backgrounds due, again, to their access to resources and having to split their focus from academic pursuits to familial or financial burdens [29]. This finding points to an increased need for scholarships that have more realistic criteria for their utilization, given that low-SES students have additional burdens to attend to while pursuing their education. 

Limitations

One limitation of this study is that we are evaluating data from a single institution. The rise in test scores could be indicative of the collaborative nature of our school’s academic culture. Additional studies at larger institutions with different student demographics will validate this study if similar results are found. Additionally, KSOM is a new institution with a limited data pool. The sample size progressively decreases from the MCAT to USMLE Step 2 (n = 240, n = 179, n = 172, n = 110, and n = 103) because KSOM is a new school, and only two classes have completed all medical school examinations. The addition of future class data would help to strengthen our finding that low-SES students are able to catch up to their peers by USMLE Step 2. Confounding variables, such as non-economic adversities, may also play a role in these results. Stratification of the data to include additional demographic information should be pursued in future studies. 

## Conclusions

Our study shows that students from low-SES backgrounds perform worse on their medical school entrance exams when compared to students from higher-SES backgrounds. This performance trend continues throughout the initial preclinical phase of their medical education. However, once they reach USMLE Step 2, they perform similarly to their peers. This trend may be due to the standardization of medical school education, regardless of SES. It is possible that earlier interventions would help to alleviate these differences before USMLE Step 2; however, careful consideration of the ramifications of the interventions should be explored. These results have implications for the admissions criteria for medical school applicants. Admissions teams are advised to explore students’ financial situations when pursuing their undergraduate degree and when studying for their MCAT exam to determine any justification for differences in performance. This data can also continue to encourage admissions teams to explore other non-academic metrics that can be used to evaluate a student’s aptitude and preparedness for the medical field. Future studies should focus on the generalizability of these results at other medical institutions as well as the impact of the proposed interventions.

## Appendices

Appendix A

The SES indicator was developed by the AAMC as a tool for medical schools to use to identify applicants who may come from a socioeconomically disadvantaged background. Information provided by applicants about their parents’ and guardians’ highest education level and occupation is used to sort applicants into five levels, EO1-EO5, using the schematic below, which is found in the American Medical College Application Service (AMCAS) Applicant Guide (Table 3). 

**Table 3:** SES indicator

Parent/Guardian Education	Parent/Guardian Occupation	
	Executive, managerial, professional position	Service, clerical, skilled, and unskilled labor
Doctorate/professional degree	SES-disadvantaged indicator: No	SES-disadvantaged indicator: Yes, EO-2
Master’s degree	SES-disadvantaged indicator: No	SES-disadvantaged indicator: Yes, EO-2
Bachelor’s degree	SES-disadvantaged indicator: No	SES-disadvantaged indicator: Yes, EO-2
Less than a bachelor’s degree	SES-disadvantaged indicator: Yes, EO-1	SES-disadvantaged indicator: Yes, EO-1

## References

[1] Grbic D, Jones DJ, Case ST (2015). The role of socioeconomic status in medical school admissions: validation of a socioeconomic indicator for use in medical school admissions. Acad Med.

[2] Benner AD, Boyle AE, Sadler S (2016). Parental involvement and adolescents’ educational success: the roles of prior achievement and socioeconomic status. J Youth Adolesc.

[3] Walpole M (2003). Socioeconomic status and college: how SES affects college experiences and outcomes. Rev High Educ. Johns Hopkins University Press.

[4] Gliatto P, Leitman IM, Muller D (2016). Scylla and Charybdis: The MCAT, USMLE, and degrees of freedom in undergraduate medical education. Acad Med.

[5] Lacour M, Tissington LD (2011). The effects of poverty on academic achievement.. https://academicjournals.org/journal/ERR/article-full-text-pdf/31F3BFB6129.

[6] Southgate E, Kelly BJ, Symonds IM (2015). Disadvantage and the 'capacity to aspire' to medical school. Med Educ.

[7] Smedley BD, Stith AY, Colburn L The right thing to do, the smart thing to do: enhancing diversity in the health professions. Symposium on diversity in health professions in Honor of Herbert W. Nickens, M.D, Washington.

[8] Griffin B, Hu W (2015). The interaction of socio-economic status and gender in widening participation in medicine. Med Educ.

[9] Hardeman RR, Burgess D, Phelan S, Yeazel M, Nelson D, van Ryn M (2015). Medical student socio-demographic characteristics and attitudes toward patient centered care: do race, socioeconomic status and gender matter? A report from the Medical Student CHANGES study. Patient Educ Couns.

[10] AAMC. 2021 FACTS: APPLICANTS AND MATRICULANTS DATA (2022). AAMC 2021 facts: applicants and matriculants data. WASHINGTON, DC: AAMC.

[11] Hill MR, Goicochea S, Merlo LJ (2018). In their own words: stressors facing medical students in the millennial generation. Med Educ Online.

[12] Greenhalgh T, Seyan K, Boynton P (2004). "Not a university type": focus group study of social class, ethnic, and sex differences in school pupils' perceptions about medical school. BMJ.

[13] Jerant A, Henderson MC, Griffin E, Talamantes E, Fancher T, Sousa F, Franks P (2018). Medical school performance of socioeconomically disadvantaged and underrepresented minority students matriculating after a multiple mini-interview. J Health Care Poor Underserved.

[14] Jerant A, Sciolla AF, Henderson MC (2019). Medical student socioeconomic disadvantage, self-designated disadvantage, and subsequent academic performance. J Health Care Poor Underserved.

[15] (2022). University of Nevada: History University of Nevada, Las Vegas. https://www.unlv.edu/medicine/history.

[16] (2023). AAMC: Graduation rates and attrition rates of U.S. medical students. https://www.aamc.org/media/48526/download.

[17] Nordli B (2022). Las Vegas Sun: Panel explores impact of proposed UNLV medical school. Sun Newspaper [Internet.

[18] Destin M, Hanselman P, Buontempo J, Tipton E, Yeager DS (2019). Do student mindsets differ by socioeconomic status and explain disparities in academic achievement in the United States?. AERA Open.

[19] Duckworth AL, Peterson C, Matthews MD, Kelly DR (2007). Grit: perseverance and passion for long-term goals. J Pers Soc Psychol.

[20] Xu KM, Meijs C, Gijselaers HJ, Neroni J, de Groot RH (2020). Measuring perseverance and passion in distance education students: psychometric properties of the grit questionnaire and associations with academic performance. Front Psychol.

[21] Pulkkinen EA, de la Ossa PP (2021). Grit and chiropractic students' academic performance: a cross-sectional study. J Chiropr Educ.

[22] Montas M, Rao SR, Atassi HA, Shapiro MC, Dean J, Salama AR (2021). Relationship of grit and resilience to dental students' academic success. J Dent Educ.

[23] Henry-Noel N, Bishop M, Gwede CK, Petkova E, Szumacher E (2019). Mentorship in medicine and other health professions. J Cancer Educ.

[24] Farkas AH, Allenbaugh J, Bonifacino E, Turner R, Corbelli JA (2019). Mentorship of US medical students: a systematic review. J Gen Intern Med.

[25] Metz AM (2017). Medical school outcomes, primary care specialty choice, and practice in medically underserved areas by physician alumni of MEDPREP, a postbaccalaureate premedical program for underrepresented and disadvantaged students. Teach Learn Med.

[26] Grumbach K, Chen E (2006). Effectiveness of University of California postbaccalaureate premedical programs in increasing medical school matriculation for minority and disadvantaged students. JAMA.

[27] AAMC. POSTBACCALAUREATE PROGRAMS DATABASE (2022). AAMC: Postbaccalaureate programs database. WASHINGTON, DC: AAMC.

[28] Andriole DA, McDougle L, Bardo HR, Lipscomb WD, Metz AM, Jeffe DB (2015). Postbaccalaureate premedical programs to promote physician-workforce diversity. J Best Pract Health Prof Divers.

[29] Baum SR, Schwartz S (1988). Merit aid to college students.. Economics of education review.

